# The Impact of Fragile X Syndrome on Caregivers: A Systematic Review

**DOI:** 10.1111/jir.70159

**Published:** 2026-08-06

**Authors:** Katerina Poprelka, Konstantina Stavrogianni, Panagiota‐Eleni Tsalouchidou, Maria Stefanatou, Anastasia Verentzioti, Athanasia Alexoudi, Anastasios Bonakis, Stylianos Gatzonis

**Affiliations:** ^1^ First Department of Neurosurgery Evangelismos Hospital, National & Kapodistrian University of Athens Athens Greece; ^2^ Second Department of Neurology National and Kapodistrian University of Athens, School of Medicine, “Attikon” University Hospital Athens Greece; ^3^ Faculty of Medicine, Department of Neurology, Epilepsy Center Hessen Marburg University Marburg Germany

**Keywords:** caregivers, family, fragile X syndrome, quality of life, unmet needs

## Abstract

**Background:**

The effects of fragile X syndrome (FXS) reach beyond the individual with the condition, profoundly influencing the well‐being of caregivers and family members. The aim of this review is to synthesise current evidence on the effects of FXS on caregivers, investigate contributors to their burden and identify gaps for future research.

**Methods:**

This review was conducted in accordance with PRISMA guidelines. A thorough search of electronic databases was performed to identify relevant original research. Two reviewers independently screened the studies for eligibility, and the quality of included studies was evaluated using the CASP tool. Key data were extracted, and a narrative synthesis was used to summarise and interpret the findings.

**Results:**

Twenty studies involving 3474 caregivers of children, adolescents and adults with FXS were included in this review. Thirteen studies were conducted in the United States, with additional research in the United States and Canada, Italy, France, the Netherlands and Australia. Female caregivers were the primary participants in most studies. Six primary factors were identified as shaping caregivers' experiences: care‐recipients' age and gender, caregivers' characteristics, disease‐related factors, compromised caregiver psychological well‐being, disrupted family dynamics and limited support systems or unmet needs. Challenging behaviours in individuals with FXS consistently emerge as the factor exerting the greatest influence on caregivers' psychological and practical burden.

**Conclusions:**

Caring for individuals with FXS places substantial burdens on caregivers, influenced by patient behaviour, family dynamics and limited support. Targeted, multidisciplinary interventions are needed to address these gaps and improve both caregiver well‐being and care outcomes.

## 1 Introduction

Fragile X syndrome (FXS) is among the most common inherited causes of intellectual disability (ID), with an estimated prevalence of about 1 in 4000–7000 males and 1 in 8000–11 000 females (Hunter et al. 2014). FXS results from an expansion of a CGG trinucleotide repeat in the *FMR1* gene on the X chromosome. Diagnostic genetic testing measures the number of CGG repeats to confirm FXS and to detect premutations that could be inherited and potentially expand in future generations. People with FXS have a full mutation, defined as more than 200 CGG repeats in the *FMR1* gene.

FXS presents with a range of neurobehavioral symptoms and conditions. The main features of FXS include ID, delays in speech and language development, autism spectrum disorder (ASD), social anxiety, mood disorders, shyness, atypical eye contact, sensory over‐responsiveness, attention‐deficit hyperactivity disorder (ADHD), aggressive behaviours, sleep disturbances, seizures, repetitive actions and obsessive‐compulsive disorder (OCD) (Genovese and Butler 2025). The clinical presentation differs by gender, with males showing higher rates of self‐injurious, aggressive and destructive behaviours compared with females, who exhibit lower rates of self‐injury and aggression (Hartley et al. 2011; Hardiman and McGill 2018).

The impact of FXS extends beyond the affected individual, significantly affecting caregivers and family members in terms of stress, emotional well‐being and overall family functioning. Caregivers play a central role in providing daily support, coordinating care and managing the complex behavioural and developmental needs associated with the condition. Biological mothers of children with FXS, in particular, may have a genetic predisposition that increases their risk for anxiety, depression and other mental health difficulties (Kenna et al. 2013; Roberts et al. 2016), whereas raising a child with FXS can make mothers more susceptible to poorer mental health outcomes. Anxiety, behavioural problems and learning difficulties in individuals with FXS are major areas of concern that place significant strain on caregivers (Weber et al. 2019). Families of children with FXS often face significant financial challenges, productivity loss and quitting employment, even when compared with families of children with ASD or ID only (Vekeman et al. 2015; Ouyang et al. 2014).

Caregivers of individuals with FXS often experience ongoing worry about their relative's future, including concerns about independence and quality of life (Feinstein and Pollack 2016). Limited support and awareness of FXS may further intensify caregiver burden (Weber 2016), whereas challenges with the transition from childhood to adulthood can create additional stress and uncertainty for families (Feinstein and Pollack 2016). Despite these significant challenges, some caregivers appear to develop resilience and coping strategies; however, evidence on these adaptive mechanisms is extremely limited in the existing literature (Feinstein and Pollack 2016; Michie and Skinner 2010).

Identifying factors that contribute to caregiver burden is essential for meeting the care needs of both individuals with FXS and their caregivers. At present, the only review examining caregivers of individuals with FXS is a scoping review of qualitative studies, highlighting common stressors, coping strategies and areas of unmet need (Kamga et al. 2020). Given the growing body of research and the critical role caregivers play in supporting individuals with FXS, a more comprehensive and up‐to‐date review is needed to expand on these findings, identify persistent gaps and inform interventions. In light of this, the present review aims to synthesise and critically evaluate existing research on the impact of FXS on caregivers, examine the factors contributing to their burden and identify directions for future research in this field.

## 2 Methods

### 2.1 Study Selection

Studies were eligible for inclusion if they involved individuals with a clinical diagnosis of FXS and their informal caregivers. Studies were eligible if they examined caregivers exclusively or included both caregivers and patients. Caregivers were defined as family members or close acquaintances who were primarily responsible for providing daily care. Only adult caregivers were considered. To capture a comprehensive picture of the impact of FXS on caregivers, studies including both paediatric and adult patients were considered. Both quantitative and qualitative research designs were included to enhance the robustness of the evidence and to gain deeper insight into caregiver experiences. Studies comparing caregiver burden across conditions were eligible for inclusion only if they provided distinct outcome data for caregivers of individuals with FXS. Studies were excluded if they did not adequately address caregivers' experiences, included paid caregivers or used unstructured methodologies. Conference proceedings, case studies, letters to the editor, dissertations, study protocols and grey literature were not considered for inclusion.

**FIGURE 1:**
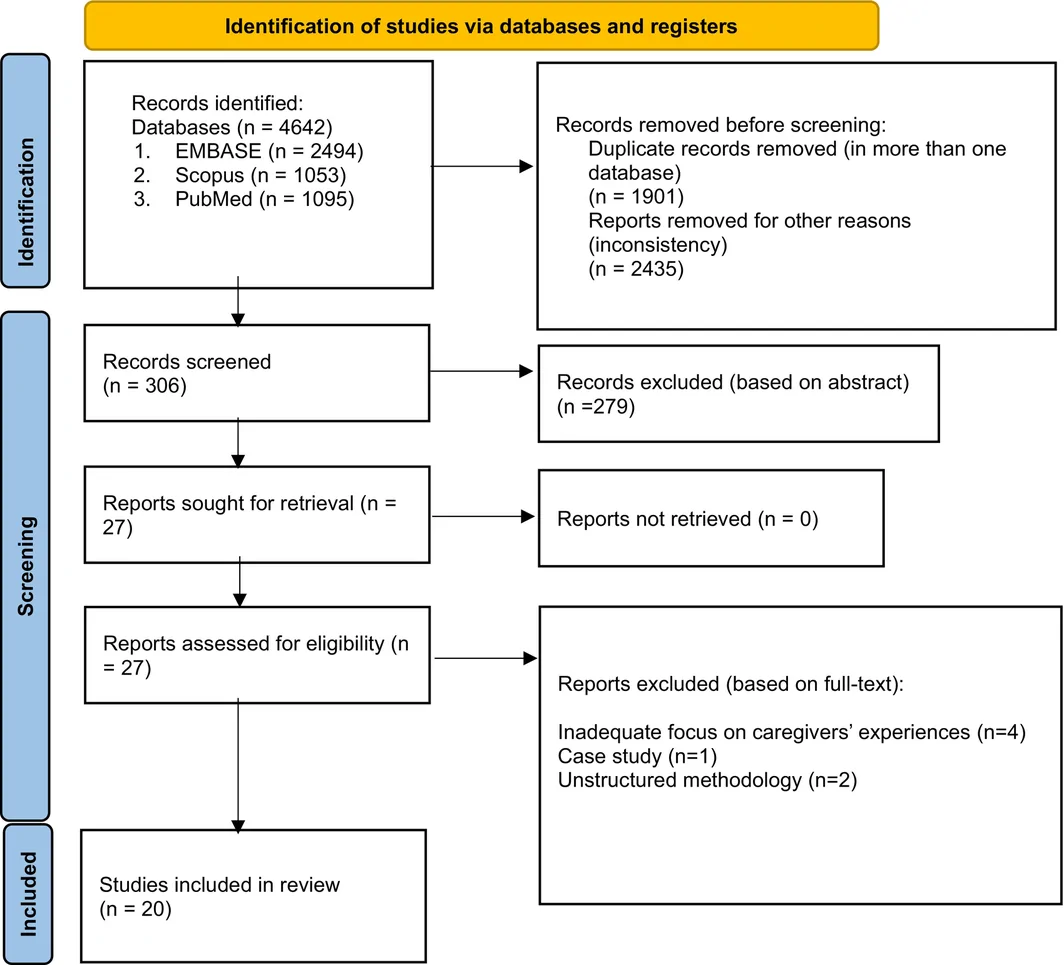
PRISMA flow diagram of identification and selection of studies related to caregiver burden in FXS.

Following PRISMA guidelines, a systematic search was conducted in EMBASE, Scopus and PubMed to identify original English‐language research articles published from January 2000 to January 2026, a period chosen to encompass recent developments in the understanding, diagnosis and caregiving of FXS. The search strategy combined terms related to FXS (‘Fragile X syndrome’, ‘Fragile X’ or FXS), caregivers (caregiver*, carer*, parent*, mother*, father* or family*) and psychosocial outcomes (stress, burden, coping, well‐being or mental health). Search strategies were adapted as required for each database. Retrieved records from all databases were imported, and duplicate records were identified and removed prior to screening. Two independent reviewers (K. P. and K. S.) independently screened the titles and abstracts of all retrieved records according to the predefined eligibility criteria. Studies considered potentially eligible underwent full‐text review to determine final inclusion. Reference lists of included studies were also manually examined for additional sources, though no further articles were identified. Although formal inter‐rater reliability statistics were not calculated, the two reviewers conducted independent screenings with minimal disagreements; any uncertainties were resolved through discussion and, when necessary, consultation with a third reviewer (S.G.) to reach consensus. The study selection process is summarised in the flow diagram shown in Figure 1.

### 2.2 Data Extraction

Data extraction was conducted using a structured Excel file. Two reviewers (K. P. and K. S.) extracted data on authors, study type (quantitative or qualitative), study design (cross‐sectional or longitudinal), patients and caregivers' characteristics, instruments used for caregivers and patients, country of origin and main findings of each study. A summary of the included studies is provided in Table 1. Given the variability and qualitative nature of the data, quantitative synthesis was not feasible. The results are therefore reported narratively, emphasising key themes and differences in caregiver experiences.

**TABLE 1:** Overview of included studies.

Author			People with FXS			Caregivers of people with FXS					
	Study type	Study design	*N*	% female	Mean age	*N*	% female	Mean age	Instruments	Country	Main findings
Abbeduto et al. (2004)	Quantitative	Cross‐sectional	22	27%	16.18	22	100%	43.52	‐ CES‐D ‐ 11‐item Pessimism subscale of the Questionnaire on Resources and Stress ‐ Positive Affect Index ‐ Carver, Scheier and Weintraub's Multidimensional Coping Inventory	United States	Mothers of individuals with FXS reported lower well‐being than mothers of children with Down syndrome but higher well‐being than mothers of children with autism, with differences varying across well‐being dimensions. The strongest predictor of maternal outcomes was the child's behavioural symptoms.
Bailey et al. (2008)	Quantitative	Cross‐sectional	108	NA	6	108	100%	35.3	‐ PSI ‐ BDI‐II ‐ STAXI‐2 ‐ Quality of Life Inventory ‐ Hope Scale ‐ LOT‐R ‐ VABS ‐ CBCL	United States	Most mothers scored in the nonclinical range on measures of stress, mood and quality of life, but nearly half showed clinically significant difficulties on at least one measure. High stress and low quality of life were most common, and child behaviour problems‐not CGG repeat length or delay severity‐significantly influenced maternal outcomes. Adaptation was multidimensional, with most mothers coping well, though a vulnerable subset was identified, particularly among those with behaviourally challenged children.
Bailey et al. (2012)	Quantitative	Cross‐sectional	350	17%	18.5	350	91%	49.9	‐ Caregiver 55‐item FXS Questionnaire ‐ ABC‐C	United States	Children averaged about five medical specialist visits per year, whereas caregivers provided 9.2 h of daily care for males with FXS, supplemented by 5.5 h of paid help. FXS had financial impacts, and caregivers took an average of 19.4 work hours per month to meet their child's needs. Nearly one‐third experienced injury from their child, with some incidents requiring medical attention. Around one‐third sought professional help for anxiety, stress or depression, and one‐fourth used medication for these symptoms. Caregiver burden was strongly linked to child problem behaviours, particularly irritability.
Baker et al. (2012)	Quantitative	Cross‐sectional	115	14%	20.46	115	100%	50.45	‐ Total Problems Scale from C/ABCL ‐ CES‐D ‐ 20‐item Marital Satisfaction Scale of the Marital Satisfaction Questionnaire ‐ Cohesion scale of the FES	United States	Behaviour problems were indirectly linked to family outcomes: they were associated with higher maternal internalising symptoms, which in turn were related to lower family cohesion and marital satisfaction. Direct effects of behaviour problems on family relationship measures were not significant.
Brady et al. (2006)	Qualitative	Cross‐sectional	55	20%	2.25	55	100%	32.2	Interviews	United States	Over half of the children were nonverbal and used augmentative or alternative communication (AAC). Mothers reported using developmentally appropriate strategies, such as reading and talking, but faced challenges in supporting communication, including limited access to speech‐language services. They described their roles as caregiver, teacher, therapist and advocate.
Chevreul et al. (2015)	Quantitative	Cross‐sectional	95	13%	19.4	69	NA	NA	‐ZBI‐22 ‐ EQ‐5D‐5L ‐ Barthel Index	France	The annual direct cost of FXS was estimated at €25, 800 per patient, primarily driven by informal caregiving and social services, with healthcare costs representing a smaller portion. Mean EQ‐5D utility scores were 0.49 for patients and 0.75 for caregivers, and the average caregiver burden was mild to moderate.
Fielding‐Gebhardt et al. (2020)	Quantitative	Longitudinal	55	20%	15.91	55	100%	46.94	‐ Positive Affect Index ‐ CBCL ‐ CES‐D ‐ Profile of Mood States	United States	The findings indicate that child challenging behaviours, maternal mental health and the quality of the mother–child relationship remained stable over time. Higher levels of child challenging behaviours were associated with poorer maternal mental health, and together these factors affected the quality of the mother–child relationship.
Hall et al. (2007)	Quantitative	Cross‐sectional	150	37.3%	10.9	150	NA	40	‐CBCL ‐SCL‐90‐R ‐FES	United States and Canada	Both children's (affected and unaffected sibling) behaviour problems had strong direct effects on maternal distress, whereas maternal distress did not influence children's behaviour. There were no differences between the effects of children with FXS and their unaffected siblings, indicating that behaviour problems in both children contributed substantially and additively to maternal depression and anxiety.
Hauser et al. (2014)	Quantitative	Longitudinal	18	22%	15.68	18	100%	46.6	‐ SCID ‐ SCL‐90‐R ‐ CED‐D ‐ Positive Affect Index ‐ Pessimism subscale from the Questionnaire on Resources and Stress ‐ FES ‐ CBCL ‐ ABC ‐ Problem Behaviour Scale from the ICAP ‐ K‐BIT	United States	Maternal mental health was not significantly associated with changes in child challenging behaviour. In contrast, child challenging behaviour was linked to changes in maternal depression over time, and higher levels of challenging behaviour were associated with increased feelings of maternal closeness toward the child over time.
Johnston et al. (2003)	Quantitative	Cross‐sectional	75	25%	10.9	75	100%	40.61	‐ PSI ‐ CBCL ‐ SCL‐90‐R ‐ FES	United States and Canada	Child behaviour, family cohesion, household income and maternal psychopathology were each differently associated with particular aspects of parenting stress. Identifying the relative influence of these factors can help guide the development of interventions aimed at improving the well‐being of mothers of children with FXS.
Jones et al. (2023)	Qualitative	Cross‐sectional	22	NA	NA	22	95%	NA	Interviews	United States	Six key themes emerged: intolerance of change and uncertainty, repetitive interests and behaviours, family impact, behavioural changes across the lifespan and the effects of the COVID‐19 pandemic. Common examples included distress over disruptions to routine, repetitive questioning, repeatedly watching the same content and caregivers needing to engage in extensive pre‐planning for everyday events.
Lanfranchi and Vianello (2012)	Quantitative	Cross‐sectional	42	5%	13.5	76	50%	44.4	‐ Questionnaire of Resources and Stress ‐ PLOC ‐ FACES‐III	Italy	Child behaviour and characteristics and parental locus of control are key factors associated with parental stress in FXS.
Lewis et al. (2006)	Quantitative	Cross‐sectional	19	0%	15.8	19	100%	43.6	‐ Positive Affective Index ‐ FES ‐ Pessimism subscale of the Questionnaire on Resources and Stress	United States	Mothers of sons with FXS experienced greater challenges to psychological well‐being than mothers of sons with Down syndrome. These differences were primarily reflected in increased concerns about their son's future and higher levels of family conflict, rather than in overall mental health impairment.
Luermans et al. (2024)	Qualitative	Cross‐sectional	22	45%	NA	11	0%	52.3	Interviews	Australia	Four themes emerged from fathers' experiences: understanding their child's condition over time, adapting to a new normal, seeking information and support and fathers' specific needs. Fathers described prolonged diagnostic journeys, post‐diagnostic grief and ongoing challenges in understanding their child's behaviours, supporting their partner, planning for the future and navigating complex care systems. They emphasised the need for father‐specific peer support and psychological counselling facilitated by health professionals.
McCarthy et al. (2006)	Quantitative	Cross‐sectional	40	22%	10.4	67	58%	40.6	‐ BSI ‐ Questionnaire on Resources and Stress ‐ BASC‐PRS ‐ DAS ‐ FSS	Australia	Parents generally showed good psychological adjustment and stable marital relationships but reported high overall family stress. Mothers and fathers experienced similar stress levels and support satisfaction, though different factors predicted their stress. Maternal stress was most strongly associated with marital satisfaction, whereas paternal stress was primarily related to the child's adaptive skills.
Ouyang et al. (2010)	Quantitative	Cross‐sectional	1019 families with at least one child with full FXS mutation	11%	NA	NA	NA	NA	Out‐of‐Pocket Cost Questionnaire (medications; therapy; respite care; supervision; genetic testing; developmental assessments; transportation; recreational needs; other costs)	United States	Nearly half of families affected by FXS reported increased financial burden, and almost 60% experienced reduced or discontinued employment due to the condition. Adequate health insurance was associated with lower perceived financial burden, whereas insurance type was not. Employment impact was greater for families of children with full mutations, particularly males, and for those with a higher number of co‐occurring conditions.
Raspa et al. (2014)	Quantitative	Cross‐sectional	1629	30%	18.1	1099	89%	46.8	4‐point scale related to the effects of FXS on seven areas of family life: parenting knowledge, social support, respondent well‐being, family social life, financial impact, overall impact of FXS and quality of life.	United States	Although families reported a generally high quality of life, they experienced challenges related to social support, social engagement and parenting knowledge. Path analysis showed that child and family characteristics influenced adaptation, whereas family resources and social supports moderated their effects on quality of life, well‐being and overall family impact.
Van Remmerden et al. (2020)	Qualitative	Cross‐sectional	5	60%	24	33	NA	NA	Interviews	Netherlands	Parents reported concerns across many areas—14 domains for males and 13 for females—including physical, psychological and socio‐economic issues. Parents in both groups experienced high stress and noted that adult care providers often lacked knowledge about FXS. The findings highlight the need for gender‐specific, multidisciplinary transitional care and ongoing adult follow‐up for individuals with FXS.
Visootsak et al. (2012)	Qualitative	Cross‐sectional	10	10%	8.5	10	100%	44.5	Interviews	United States	Three key issues for African American families affected by FXS were found: Testing is often delayed after parents first raise developmental concerns, diagnoses are sometimes delivered insensitively with outdated and overly negative information and families experience communication difficulties after learning the diagnosis. These findings highlight important needs for improved genetic counselling, support and advocacy.
Wheeler et al. (2008)	Mixed‐Methods	Cross‐sectional	101	14%	6.5	101	100%	36	‐Interviews ‐ The Quality of Life Inventory ‐ SCID ‐ State Hope Scale and Trait Hope Scale ‐ PSI ‐ FSS ‐ PAIR Inventory ‐ Fewell Religion Scale	United States	Mothers of children with FXS reported overall quality of life similar to population norms. Trait hope and parenting stress were significant predictors of quality of life. Although many viewed motherhood as a major positive influence, they also experienced notable challenges and stress, especially when lacking support from their social environment.

### 2.3 Quality Assessment

The quality of the included studies was assessed using the Critical Appraisal Skills Programme (CASP). The CASP Cross‐Sectional Study Tool was applied to cross‐sectional studies, the CASP Cohort Study Tool to longitudinal studies and the CASP Qualitative Research Tool to qualitative studies. Each study was independently evaluated by two reviewers (K.P. and P.‐E.T.). Although formal percent agreement was not calculated, discrepancies were minimal and resolved through discussion and consensus. Following previous studies (Bowden et al. 2023), each study's appraisal score was expressed as the percentage of applicable criteria met. Studies scoring below 60% were considered low quality, those scoring 60%–79% as fair quality and those scoring 80% or higher as high quality.

## 3 Results

### 3.1 Studies Characteristics

Twenty studies investigating the impact of FXS on caregivers were included in the review. Fourteen studies applied quantitative designs, five used qualitative approaches and one adopted mixed‐methods design. Thirteen studies were conducted in the United States, two across both the United States and Canada and one each in Italy, France and the Netherlands, with two additional studies carried out in Australia. All studies were cross‐sectional, with the exception of two that used longitudinal designs. The majority of studies included mainly female caregivers, with one study dedicated to investigating fathers' experiences (Luermans et al. 2024). On the other hand, most studies reported a predominance of male patients, except for one (Van Remmerden et al. 2020).

Three studies included paediatric patients (Brady et al. 2006; Hall et al. 2007; Wheeler et al. 2008), whereas seven studies included both paediatric and adolescent participants (Abbeduto et al. 2004; Bailey et al. 2008; Fielding‐Gebhardt et al. 2020; Johnston et al. 2003; Lewis et al. 2006; McCarthy et al. 2006; Visootsak et al. 2012). One study included adolescents and adults (Baker et al. 2012), and one study focused exclusively on adults (Van Remmerden et al. 2020). The remaining studies included participants across the lifespan. Some studies provided information regarding the mutation or premutation status of biological mothers as caregivers. Two studies included mothers carrying the full mutation (Brady et al. 2006; Wheeler et al. 2008), three included mothers with the premutation (Baker et al. 2012; Hall et al. 2007; Johnston et al. 2003) and five reported samples comprising both groups (Bailey et al. 2008; Fielding‐Gebhardt et al. 2020; Hauser et al. 2014; Lewis et al. 2006; McCarthy et al. 2006). Due to the absence of standardised tools for assessing caregiver burden, quality of life and the psychosocial impact of FXS, studies employed a variety of different measures.

### 3.2 Results of Quality Assessment

The quality of the included studies was rated as ‘high’ in eighteen studies (Bailey et al. 2008; Bailey et al. 2012; Baker et al. 2012; Brady et al. 2006; Chevreul et al. 2015; Fielding‐Gebhardt et al. 2020; Hall et al. 2007; Hauser et al. 2014; Jones et al. 2023; Johnston et al. 2003; Lewis et al. 2006; Luermans et al. 2024; McCarthy et al. 2006; Ouyang et al. 2010; Raspa et al. 2014; Van Remmerden et al. 2020; Visootsak et al. 2012; Wheeler et al. 2008) and ‘fair’ in two studies (Abbeduto et al. 2004; Lanfranchi and Vianello 2012). No studies were excluded based on quality to allow for the inclusion of all potentially relevant findings. Tables S1, S2 and S3, summarising the quality assessment criteria for cross‐sectional, longitudinal and qualitative studies, respectively, are available in the Supporting Information.

### 3.3 Demographics of Care Recipients

Ten studies investigated the influence of patients' gender and age on caregivers. Four studies reported that neither patients' age nor gender significantly influenced caregiver experiences or outcomes (Chevreul et al. 2015; Baker et al. 2012; Fielding‐Gebhardt et al. 2020; Johnston et al. 2003). On the other hand, the study by Bailey et al. (2012) found that male patients required greater daily caregiving time than female patients and were associated with a higher likelihood of caregiver injuries requiring medical treatment. Similarly, a study in the Netherlands showed that male patients had higher levels of ID and required daily care and support (Van Remmerden et al. 2020). Families with two or more male children carrying the full mutation were nearly twice as likely to report that a family member had left employment compared with families with only affected female children, and families of male patients were also more likely to experience negative economic impacts than those of female patients (Ouyang et al. 2010). A negative correlation has been observed between child's age and family problems for both mothers and fathers (Lanfranchi and Vianello 2012), whereas fathers' physical burden increased with child age, with older children associated with greater physical strain (McCarthy et al. 2006). Families with an affected child under 20 years of age were more likely to experience a financial burden (Ouyang et al. 2010), whereas families with younger children reported worse adaptation (Raspa et al. 2014). No other demographic characteristics of patients have been examined in relation to their impact on caregivers in the included studies.

### 3.4 Caregivers' Characteristics

Evidence indicates that caregiver demographics can affect overall well‐being. Most studies reported a predominance of female caregivers, except for one that focused on fathers' experiences (Luermans et al. 2024). Only a small number of studies have explored differences in burden experienced by mothers versus fathers. One study demonstrated that caregiver burden did not differ between mothers and fathers (Chevreul et al. 2015). Similarly, the studies by Lanfranchi and Vianello (2012) and McCarthy et al. (2006) found that the overall impact of FXS was comparable for mothers and fathers; however, different factors predicted caregiver well‐being based on gender. Fathers face difficulties in addressing their child's needs, supporting their partner, planning ahead and managing complex care systems (Luermans et al. 2024), whereas mothers report high parenting stress and depression (Johnston et al. 2003; Baker et al. 2012). Additionally, compared with mothers, fathers have been found to report lower levels of parenting knowledge (Raspa et al. 2014). Mothers commonly take on multiple roles in caring for their children, often balancing caregiving and advocacy for their child's needs (Brady et al. 2006). According to one study, caregivers' age did not significantly correlate with the levels of burden (Chevreul et al. 2015).

Findings on the impact of caregiver education are mixed. Evidence on maternal education was mixed, with higher education associated with lower depression in one study (Baker et al. 2012) but linked to reduced hope in another (Wheeler et al. 2008). In contrast, no relationship was observed between parents' education and quality of life (McCarthy et al. 2006). Similarly, occupation was not associated with parents' quality of life in one study (McCarthy et al. 2006); however, other authors do not corroborate these findings. Caregivers reported adjusting work hours, taking extended absences, leaving their jobs, changing positions or declining promotions to care for a relative with FXS (Bailey et al. 2012; Ouyang et al. 2010). Caring for younger patients or those with more co‐occurring conditions may further impact caregivers' employment (Ouyang et al. 2010). Of note, being in a good work environment contributes to higher quality of life (Wheeler et al. 2008).

Reduced work hours may intensify financial strain, adding to the economic challenges faced by families of individuals with FXS. Lower income has been associated with greater depression, anxiety, feelings of acceptability and lower quality of life in mothers taking care of children with FXS (Abbeduto et al. 2004; Baker et al. 2012; Johnston et al. 2003; Wheeler et al. 2008). Two studies conducted in the United States found that only 21% and 27% of families, respectively, reported that having a child with FXS did not result in a financial burden (Bailey et al. 2012; Raspa et al. 2014). Furthermore, families earning $50 001–$100 000 are less likely to experience parental job loss or report financial strain (Bailey et al. 2012). Another study revealed that transportation and therapy accounted for over 30% of total costs (Ouyang et al. 2010). On the other hand, having sufficient finances contributes to higher levels of quality of life (Wheeler et al. 2008). Finally, a study conducted in France found that the total annual direct cost associated with FXS was estimated at €25 800 per patient (Chevreul et al. 2015). Together, these results emphasise that financial resources significantly influence both the economic impact of FXS and the well‐being of caregivers.

### 3.5 Disease‐Related Factors

The clinical features of FXS can significantly affect individuals who care for those living with the condition. The diagnostic journey can place considerable emotional and practical strain on caregivers. Parents report a complex diagnostic journey before their child received a genetic diagnosis of FXS, involving consultations with multiple specialists and numerous tests (Luermans et al. 2024). Additionally, limited physician awareness of FXS was identified as a barrier to timely diagnosis (Visootsak et al. 2012).

Dealing with behavioural symptoms may considerably affect caregivers' daily routines and quality of life. The majority of the included studies explored how behavioural symptoms affect caregivers. Challenging behaviours, such as irritability, caregiver injuries and inflexibility, significantly impact caregivers' psychological well‐being and quality of life (Bailey et al. 2008; Bailey et al. 2012; Baker et al. 2012; Jones et al. 2023; Abbeduto et al. 2004; Wheeler et al. 2008). Children's challenging behaviours have been associated with poorer mother–child relationship quality and worse maternal mental health (Fielding‐Gebhardt et al. 2020). Maternal feelings of competence and acceptability toward the child have also been associated with behavioural symptoms (Johnston et al. 2003). Interestingly, a longitudinal study by Hauser et al. (2014) indicated that higher rates of child challenging behaviours were related to a decrease in maternal symptoms of depression, suggesting a more complex relationship between child behaviour and maternal mental health.

Notably, Baker et al. (2012) found that behavioural symptoms did not significantly affect family relationships, whereas Hall et al. (2007) reported that problem behaviours in children with FXS did not appear to contribute to greater maternal distress compared with the behaviours of unaffected siblings. In contrast, mothers of sons with co‐morbid FXS and autism experienced greater family conflict and reduced closeness compared with mothers of children with FXS only or Down syndrome (Lewis et al. 2006). Although behavioural difficulties affect both mothers and fathers, McCarthy et al. (2006) found that such problems did not significantly predict maternal stress, whereas paternal mental health was significantly influenced by children's externalising behaviours and adaptive functioning.

Regarding patients' cognitive functioning, findings are mixed, with some studies reporting a significant impact on caregiver burden, whereas others found no clear association. Child's IQ and cognitive deficits did not appear to influence mothers' well‐being in two studies (Hall et al. 2007; Johnston et al. 2003). On the other hand, children's learning disabilities and communication challenges have been associated with a higher number of paid caregiving hours and greater maternal concern (Bailey et al. 2012; Brady et al. 2006). Finally, one study highlighted that caregivers faced difficulties during the transition to adult care, citing insufficient referrals, limited communication between paediatric and adult physicians and a lack of support that required them to manage the process independently (Van Remmerden et al. 2020).

### 3.6 Caregivers' Psychological Well‐Being

The psychological impact of caring for a child with FXS can be substantial. Caregivers often face increased emotional burden and mental health challenges. The majority of the included studies focused on maternal psychological well‐being. Depression, anxiety and stress affect a significant percentage of caregivers (Bailey et al. 2008; Johnston et al. 2003; Raspa et al. 2014; Van Remmerden et al. 2020; Wheeler et al. 2008), highlighting the considerable psychological strain associated with caring for a child with FXS. A longitudinal study indicated that maternal depression, anxiety and anger tend to remain stable over time (Fielding‐Gebhardt et al. 2020), highlighting the enduring nature of emotional challenges faced by caregivers. Interestingly, another longitudinal study found that higher rates of challenging behaviours were associated with decreased maternal depression over time (Hauser et al. 2014), suggesting a more complex relationship between child behaviour and maternal psychological well‐being. Literature suggests that biological mothers carrying the premutation may experience elevated levels of stress, anxiety and depressive symptoms, potentially affecting their overall functioning; however, findings regarding the association between premutation status and depressive symptoms remain inconsistent, with some studies reporting a positive association (Bailey et al. 2008; Fielding‐Gebhardt et al. 2020), whereas others have not corroborated these findings (Baker et al. 2012).

Although caregivers often report feelings of pessimism, grief and concern about the future (Lewis et al. 2006; Luermans et al. 2024; Wheeler et al. 2008), many also experience hope and optimism, which have been associated with better quality of life (Bailey et al. 2008; Wheeler et al. 2008). Only a few included studies have explored coping strategies among caregivers of individuals with FXS. Mothers who relied on problem‐focused coping strategies and avoided emotion‐focused coping reported experiencing greater closeness with their child (Abbeduto et al. 2004), whereas others reported that their child's disability did not hold them back; instead, it motivated them to seek services, advocate for their child, engage them in social and community activities and remain socially active themselves (Wheeler et al. 2008). Effective coping and a positive, engaged approach appear to support caregiver well‐being and strengthen the parent–child bond.

### 3.7 Family Dynamics

Living with FXS can influence multiple aspects of family functioning, including family relationships, roles and overall family dynamics, with caregivers often experiencing significant adjustments in their daily lives and responsibilities. Caregivers often have to modify routines, family activities and social engagements to accommodate the child's needs (Jones et al. 2023), especially when children are severely affected (Raspa et al. 2014). Cohesiveness of the family reduces parental stress and isolation (Johnston et al. 2003), whereas lack of understanding and support from family members may further exacerbate caregivers' strain and reduce their quality of life (Luermans et al. 2024; Visootsak et al. 2012; Wheeler et al. 2008). A study highlighted that families may not share the diagnosis of FXS with immediate and extended family members, which can delay or prevent the identification and diagnosis of other at‐risk relatives, limiting early intervention and access to appropriate care (Visootsak et al. 2012).

Marital satisfaction has also been linked to caregiver well‐being, as supportive partner relationships may buffer stress and promote better psychological adjustment. Three studies support that greater marital satisfaction reduces maternal depression and anxiety (Baker et al. 2012), promotes their mental health (McCarthy et al. 2006) and improves their quality of life (Wheeler et al. 2008). Divorced mothers tend to report more parenting stress, depression and less family support compared with single or married ones (Wheeler et al. 2008). On the other hand, fathers report that caring for a child with FXS may lead to marital strain; however, they also describe maintaining strong relationships within the nuclear family (Luermans et al. 2024). Fathers described an added layer of complexity related to their spouse's feelings of guilt about passing on FXS, which often made them reluctant to express their own emotions and left some feeling ill‐equipped to adequately support their partner without guidance (Luermans et al. 2024).

Having more than one child with disability may further affect family dynamics and decrease psychological health (Abbeduto et al. 2004). Caregivers with more than one affected child reported that FXS impacted each child differently, and emphasised the importance of recognising each child's individuality, including understanding behavioural triggers and identifying activities the child enjoys (Luermans et al. 2024). Caregivers are also concerned that siblings without FXS may miss out on certain experiences in order to accommodate the needs and comfort of the child with FXS (Jones et al. 2023), whereas the family environment seems to significantly influence the behaviours of the unaffected children (Hall et al. 2007). Finally, families who possess greater knowledge about FXS tend to experience lower financial burdens, likely because informed families can better navigate available supports and services (Ouyang et al. 2010). Additionally, families with more severely affected children or greater access to resources often acquire higher levels of understanding about FXS and the range of interventions and assistance programs, enabling them to make more informed decisions and optimise care for their child (Raspa et al. 2014).

### 3.8 Support Systems and Unmet Needs

Caregivers of individuals with FXS often rely on formal and informal support systems to help manage the demands of caregiving, including family members, friends, healthcare professionals and community resources. Social support and better social lives have been related to higher quality of life and better well‐being (Raspa et al. 2014). Nevertheless, it is common for caregivers to experience limited support and understanding from their social and professional networks (Luermans et al. 2024). A proportion of caregivers, especially mothers, experience social isolation, which can adversely impact their mental health (Johnston et al. 2003). Notably, a study reported that social support did not significantly predict levels of stress among parents (McCarthy et al. 2006), suggesting that other factors may play a more critical role in determining parental stress.

Caregivers of individuals with FXS often report unmet needs, although only a few studies included in this review investigated them. Caregivers described experiencing at least one negative encounter with clinical services, including perceived inadequate treatment of their child or family by healthcare professionals, limited or outdated knowledge about FXS among medical staff and restricted access to available services, especially in regional and remote areas (Luermans et al. 2024; Visootsak et al. 2012).

A study in the Netherlands (Van Remmerden et al. 2020) reported that parents of individuals with FXS were concerned about recent financial cuts in the healthcare system and found the process of applying or reapplying for a personal budget to be complex and frustrating, often feeling distrusted by government officials. Parents of female patients expressed additional concerns that their daughters frequently did not qualify for personal budgets, social security benefits or care reimbursements due to normal intelligence levels. Families also reported a lack of knowledge about FXS among physicians and other healthcare providers, highlighting the need for a multidisciplinary treatment centre for adult patients that could support local providers and offer appropriate living facilities. Many parents described the transition to adult care as insufficient, with no referrals or communication between paediatric and adult services, leaving them to navigate care on their own and report a lack of adequate support.

## 4 Discussion

Caring for individuals with FXS can be a demanding and overwhelming responsibility. This review sheds light on the complex experiences of caregivers, emphasising the multifaceted challenges they encounter. Factors such as care‐recipients' demographics, caregivers' own characteristics, disease‐related variables, compromised caregiver psychological well‐being, disrupted family dynamics and limited support systems have all been identified as significant correlates to caregiver burden. These findings underscore the profound impact of FXS on both patients and caregivers, highlighting the need for comprehensive, multidisciplinary care and support strategies.

Several studies included in the review found no significant association between these patients' gender and caregiver experiences. However, some evidence suggests male patients may be associated with greater caregiving demands, including increased daily care time, higher risk of caregiver injury and greater economic impact on families. This may partly reflect the higher prevalence of challenging behaviours among males, who have been reported to exhibit higher levels of self‐injurious, aggressive and destructive behaviours, whereas females tend to demonstrate lower rates of both self‐injury and aggression (Hardiman and McGill 2018). Furthermore, females with FXS tend to demonstrate higher functional skills compared with males (Bailey et al. 2009), which may contribute to lower caregiving demands and reduced caregiver burden compared with families of affected males. Sex‐based differences may be largely attributed to the X‐linked inheritance pattern of FXS, whereby males typically present with more severe cognitive and behavioural impairments due to the presence of a single X chromosome, whereas females often exhibit milder or more variable clinical manifestations as a result of X‐chromosome inactivation (Bartholomay et al. 2019). Nevertheless, these differences should be interpreted with caution, as females with FXS represent a heterogeneous population with a broad range of cognitive, behavioural and functional outcomes. A subset of females may experience substantial impairments and support needs comparable with those observed in males; however, their limited representation in existing studies may not adequately reflect the full spectrum of female presentations.

Similarly, the findings regarding the impact of patients' age on caregiver experiences are mixed. Some studies suggest that caregivers of younger children with FXS face greater stress and financial burden, likely due to higher daily care demands and challenges in adaptation, whereas other studies indicate that caregiver strain may increase as children grow older, particularly for fathers, due to greater physical demands or behavioral challenges associated with adolescence and adulthood (Lanfranchi and Vianello 2012; McCarthy et al. 2006; Raspa et al. 2014). These mixed findings may be partly explained by the fact that challenging behaviours in individuals with FXS can emerge at any age, meaning that caregiver demands are not confined to a specific developmental stage (Abbeduto et al. 2019; Hagerman and Hagerman 2002). Another explanation for the mixed findings is that both younger and older ages present unique challenges for caregivers. Early caregiving often involves significant emotional stress and adjustment to the child's diagnosis, whereas later stages may place greater demands on caregivers due to intensive, long‐term care needs. These patterns may suggest a U‐shaped relationship between age and caregiver burden, with peaks during early childhood and later adulthood and a period of relative stabilisation between these stages. Similar to typical developmental transitions, periods of early childhood and adolescence may represent times of increased adjustment and caregiving demands; however, in FXS, these challenges are likely experienced at a higher intensity and may persist longer due to lifelong support requirements.

In line with the findings of this review, caregiving responsibilities are predominantly assumed by females, with mothers typically serving as the primary caregivers (Kamga et al. 2020). In fact, nearly all studies included in this review noted a clear predominance of female participants in caregiving roles. This finding is reported in other chronic illnesses across the lifespan, such as Prader–Willi Syndrome (Usubini et al. 2024), Down syndrome (Bodde et al. 2025; Chiracu et al. 2023), autism (Daharlı et al. 2025), epileptic and developmental encephalopathies (Gallop et al. 2010; Poprelka, Fasilis, Patrikelis, Ntinopoulou, et al. 2025) and tuberous sclerosis (Willems et al. 2021). Female caregivers, especially mothers, often assume multiple roles, balancing direct care, household responsibilities and coordination of medical and educational services, which may increase stress, fatigue and risk of mental health difficulties. Moreover, the overrepresentation of female caregivers in research highlights a gap in understanding the experiences of fathers and other male caregivers, who may face unique challenges and coping needs that remain underexplored.

Caring for a child with FXS can substantially affect caregivers' employment and financial stability, with many parents adjusting work hours, taking leave or leaving their jobs. Consistent with the findings of this review, a study on the employment and financial impact for families of children with FXS, ASD and ID found that 40% of parents of children with FXS and 46% of parents of children with both ASD and ID had quit their jobs due to caregiving responsibilities (Ouyang et al. 2014). These disruptions to employment contribute to a significant financial burden, as families often face increased out‐of‐pocket costs for therapies, transportation and specialised services. Several studies have indicated that caring for a child with FXS imposes a substantial economic burden. For example, studies in the United States and Europe indicate that families face high direct costs for therapies, medical care and support (Vekeman et al. 2015). Even families with moderate incomes may experience financial strain, highlighting the widespread economic impact of FXS on households and the critical need for adequate financial support and accessible services.

The literature consistently demonstrates that behavioural symptoms in FXS contribute substantially to caregiver burden. Most of the included studies examined the impact of behavioural symptoms on caregivers, with findings consistently indicating a significant contribution to caregiver strain and reduced well‐being. Most studies found a negative impact of challenging behaviours on caregivers; however, some findings in the literature suggest a more complex and context‐dependent relationship. For instance, the longitudinal study by Hauser et al. (2014) reported that higher rates of child challenging behaviours were associated with a decrease in maternal depressive symptoms. One possible explanation is that mothers who are more actively engaged in managing their child's behavioural difficulties may develop coping strategies, increased resilience or a greater sense of competence over time, which could buffer depressive symptoms. Additionally, longitudinal designs may capture adaptation processes that are not evident in cross‐sectional studies. Similarly, Baker et al. (2012) found that behavioural symptoms did not significantly affect family relationships, whereas Hall et al. (2007) reported that problem behaviours in children with FXS did not lead to greater maternal distress compared with the behaviours of unaffected siblings. These findings may be partly explained by parental adaptation or resilience to the child's behavioural profile, particularly in families who have lived with the condition for several years. In such contexts, caregivers may normalise or reinterpret challenging behaviours as part of the child's developmental condition, thereby reducing their perceived psychological impact. Nevertheless, evidence from studies of FXS and other genetic conditions supports the notion that behavioural symptoms contribute an additional layer of burden for caregivers (Muller et al. 2019; Patel et al. 2018; Poprelka, Fasilis, Patrikelis, Stefanatou, et al. 2025; Sadhwani et al. 2019; Domaradzki and Walkowiak 2024), as these symptoms are often associated with increased caregiving demands, higher levels of stress and greater emotional strain.

Caring for a child with FXS can have a substantial psychological impact on caregivers, particularly mothers. Although fathers were included in only a limited number of studies, findings indicate that they, too, experience compromised psychological well‐being (Luermans et al. 2024; McCarthy et al. 2006). Literature suggests that caregivers in FXS frequently face psychological distress, including feelings of grief and loss, anxiety and depression, while also overlooking their personal health care (Iosif et al. 2013; Kamga et al. 2020; Bullard et al. 2021). Similarly, caregivers of individuals with chronic conditions, including genetic and neurodevelopmental disorders, have been shown to experience poorer mental health (Mohandas et al. 2021; Maridal et al. 2021). Moreover, biological mothers who carry the FMR1 premutation may be at increased risk for mental health difficulties, such as anxiety, depression and stress, suggesting a potential genetic contribution to caregiver psychological vulnerability (Bourgeois et al. 2011; Gossett et al. 2016). Some caregivers, particularly mothers who carry the FMR1 premutation, may also experience feelings of guilt for having passed FXS on to their children. However, this emotional burden has not been extensively studied, and little is known about its prevalence or impact on caregiver mental health. The lack of empirical evidence highlights an important gap in the literature, underscoring the need for research that explores genetic‐related guilt and its potential psychological consequences.

Although coping strategies are recognised as important for supporting caregiver well‐being in FXS, the existing evidence is limited and largely descriptive. Some studies suggest that mothers who rely on problem‐focused coping and actively engage with their child's needs report closer parent–child relationships and greater psychological resilience (Abbeduto et al. 2004). Others indicate that caregiving challenges can motivate advocacy, participation in social and community activities and proactive engagement with services (Wheeler et al. 2008). Despite these insights, few studies have systematically examined which specific coping strategies are most effective or how they interact with caregiver mental health over time. This represents a critical gap in understanding the mechanisms through which coping strategies function as protective factors, highlighting the need for targeted interventions that strengthen adaptive coping and foster resilience among caregivers of individuals with FXS.

It is well recognised that having a family member with a chronic condition, such as FXS, can substantially increase stress within the household, often leading to strained relationships, altered family roles and decreased overall cohesion. Families that demonstrate strong adaptability and effective parental coping tend to experience more positive psychological outcomes, whereas disruptions in family functioning are linked to greater emotional and behavioural challenges and lower adherence to medical treatments. Furthermore, some families do not openly discuss FXS, and in several cases, participants were unaware that extended family members had previously been diagnosed with either the premutation or full mutation. Even when FXS is discussed, there is frequently misinformation regarding which family members were at risk (Visootsak et al. 2012). These communication gaps and misunderstandings may contribute to confusion, increased anxiety and challenges in family planning and caregiving within affected families. On the other hand, evidence suggests that families with strong informal support networks, such as friends and extended family, generally experience more positive outcomes (Ekas et al. 2010). Access to formal support from therapists or other professionals has also been shown to enhance parental well‐being (Trivette et al. 2010). These findings underscore the potential benefits of connecting families with both peer networks and formal services, which may help buffer the challenges of caregiving and promote resilience in the face of adversity.

Caregivers of individuals with chronic conditions frequently face unmet needs related to emotional support, access to services, financial assistance and care coordination. These challenges are amplified in rare diseases such as FXS, where the complexity of care and lack of tailored resources increase caregiver burden. Families caring for individuals with FXS often report limited guidance from healthcare providers, inadequate access to mental health and respite services and poor awareness of FXS among professionals, leaving caregivers to manage coordination of care themselves. Financial support is limited and difficult to navigate, whereas the transition from paediatric to adult services adds further strain due to the absence of structured pathways, potentially leading to missed interventions and increased emotional stress. Fragmented care and under‐resourced adult services further exacerbate these challenges, although caregivers value when emotional support is provided. Despite the long‐term and complex nature of caregiving in FXS, targeted interventions for caregivers remain scarce. Caregiver‐focused programs, such as psychoeducation and counselling, have not been systematically implemented in FXS, highlighting a critical gap. Addressing this requires a shift in care models to prioritise caregiver well‐being alongside patient management. Integrating structured, evidence‐based interventions into multidisciplinary FXS care could reduce caregiver burden, enhance mental health and improve overall care stability, drawing lessons from other chronic neurological and genetic conditions, such as developmental and epileptic encephalopathies, autism, Prader–Willi syndrome and Williams syndrome, where such programs have demonstrated positive outcomes (Nevin et al. 2022; Baroi and Muhammad 2025; Bedard et al. 2025; Graham et al. 2025). Genetic counselling should extend beyond the provision of genetic risk information to incorporate routine assessment of patients' and families' psychosocial needs, facilitate informed decision‐making and provide ongoing emotional support throughout the disease trajectory. Future research should focus on developing and evaluating tailored supports that address the emotional, psychological and practical needs of caregivers.

### 4.1 Limitations

This review has several limitations that should be considered when interpreting its findings. Many studies did not use validated tools specifically designed to assess caregiver burden and psychosocial impact in FXS, which, while allowing for the inclusion of a broader range of research, may have introduced variability in measurement approaches and limited the reliability and comparability of results. The inclusion of studies involving children, adolescents and adults with FXS provided a more comprehensive perspective on caregiver burden across the lifespan, but it may also have introduced heterogeneity due to the different challenges caregivers face at various life stages. Several studies had relatively small samples and were predominantly composed of female caregivers, which may limit generalisability and underrepresent the experiences of male caregivers. Maternal neurocognitive status was not measured in the studies included in this review, despite its potential influence on maternal perceptions of the child and, indirectly, maternal well‐being. Only English‐language, peer‐reviewed studies were considered to ensure methodological rigor, yet this approach may have introduced publication bias and excluded relevant research published in other languages or in grey literature. Most studies were cross‐sectional, restricting understanding of how caregiver burden evolves over time. Finally, all included studies were conducted in the United States, Europe and Australia, limiting insight into caregivers' experiences in other regions and potentially overlooking cultural, social and healthcare system differences that could shape caregiver burden in African and Asian contexts.

### 4.2 Future Directions

Future research should focus on several key areas to better understand the impact of FXS on caregivers. Studies should include underrepresented caregiver groups, such as males and individuals from diverse socioeconomic and ethnic backgrounds, to provide a more complete picture of caregiver burden. The inclusion of female patients across the full spectrum of disease severity and functional impairment should also be prioritised. This would improve the representativeness and generalisability of findings while fostering a more comprehensive understanding of the diverse experiences and support needs of patients and their families. Cross‐cultural research is needed to account for differences between Western and non‐Western contexts and to inform the development of culturally appropriate support systems. Priority should be given to creating and validating tools specifically designed to assess caregiver burden and quality of life in FXS, ensuring more reliable and standardised measurements. Additional research is needed on caregivers' coping strategies, resilience and experiences of stigma, which remain underexplored. Caregiver feelings of guilt associated with the genetic transmission of FXS have not been sufficiently investigated in the existing literature. The shift from paediatric to adult services is a major challenge, but there is limited research on how it affects caregivers. Longitudinal studies are essential to capture how caregiver strain evolves over time as the needs of individuals with FXS change across the lifespan. Including comparison groups, such as caregivers of individuals with other rare syndromes, could help clarify the unique challenges associated with FXS. Moreover, greater emphasis should be placed on the development, implementation and evaluation of psychoeducational programs and tailored interventions that address caregivers' specific needs. Such initiatives have the potential to reduce caregiver stress and burden, enhance coping strategies and self‐efficacy and ultimately support the provision of more comprehensive and holistic care. Finally, healthcare policies and financial assistance programs should be systematically assessed for their effectiveness in alleviating caregiver burden and improving family quality of life.

## 5 Conclusion

In summary, caring for individuals with FXS places substantial emotional, physical and financial demands on caregivers, particularly mothers. Caregiver burden is influenced by multiple factors, including patient characteristics, behavioural challenges, disrupted family dynamics, limited social and professional support and genetic vulnerability such as maternal premutation status. Although coping strategies, family adaptability and social support can mitigate some of these stresses, the current evidence highlights significant gaps, including underrepresentation of fathers, limited research on genetic‐related guilt and a lack of structured, caregiver‐focused interventions. Addressing these gaps through multidisciplinary care models that incorporate psychological, social and practical supports for caregivers is essential. Targeted interventions and systemic support have the potential not only to improve caregiver well‐being but also to enhance overall care quality and outcomes for individuals with FXS.

## Funding

The authors have nothing to report.

## Ethics Statement

There are no human participants in this article, and informed consent is not required.

## Conflicts of Interest

The authors declare no conflicts of interest.

## Artificial Intelligence (AI) Statement

The authors declare that no artificial intelligence (AI) tools were used in the preparation of this manuscript.

## Supporting information




**Table S1:** Quality assessment of cross‐sectional studies using CASP Cross‐Sectional Study Tool.
**Table S2:** Quality assessment of the longitudinal study using CASP Cohort Study Tool.
**Table S3:** Quality assessment of the qualitative studies using CASP Qualitative Research Tool.

## Data Availability

All data relevant to this study are included in the article and in the supporting information.
