# Supplementary material for: The Impact of Fragile X Syndrome on Caregivers: A Systematic Review
Source: J Intellect Disabil Res. 2026 Aug 6;70(9):945–61. doi: 10.1111/jir.70159 (PMC13537334; doi:10.1111/jir.70159)
Supplement: Supplementary file 1 [file JIR-70-945-s001.docx]

**Supplementary Tables**

**Table S1.** Quality assessment of cross-sectional studies using CASP Cross-Sectional Study Tool

| Study | Q1 | Q2 | Q3 | Q4 | Q5 | Q6 | Q7 | Q8 | Q9 | Q10 | Q11 | Total Quality  Assessment | |  |
| --- | --- | --- | --- | --- | --- | --- | --- | --- | --- | --- | --- | --- | --- | --- |
| Abbeduto  et al. (2004) | **Y** | **Y** | **CT** | **Y** | **CT** | **N** | **Y** | **Y** | **Y** | **CT** | **Y** | | **64%** | |
| Bailey et al.  (2008) | **Y** | **Y** | **CT** | **Y** | **Y** | **Y** | **Y** | **Y** | **Y** | **Y** | **Y** | | **90%** | |
| Bailey et al.  (2012) | **Y** | **Y** | **CT** | **Y** | **Y** | **Y** | **Y** | **Y** | **Y** | **Y** | **Y** | | **90%** | |
| Baker et al.  (2012) | **Y** | **Y** | **CT** | **Y** | **Y** | **Y** | **Y** | **Y** | **Y** | **CT** | **Y** | | **82%** | |
| Chevreul et al.  (2015) | **Y** | **Y** | **Y** | **Y** | **Y** | **Y** | **Y** | **Y** | **Y** | **Y** | **Y** | | **100%** | |
| Hall et al.  (2007) | **Y** | **Y** | **CT** | **Y** | **Y** | **Y** | **Y** | **Y** | **Y** | **CT** | **Y** | | **82%** | |
| Johnston et al.  (2003) | **Y** | **Y** | **Y** | **Y** | **Y** | **Y** | **Y** | **Y** | **Y** | **CT** | **Y** | | **90%** | |
| Lanfranchi &  Vianello (2012) | **Y** | **Y** | **CT** | **Y** | **CT** | **CT** | **Y** | **Y** | **Y** | **CT** | **Y** | | **64%** | |
| Lewis et al.  (2006) | **Y** | **Y** | **Y** | **Y** | **Y** | **N** | **Y** | **Y** | **Y** | **CT** | **Y** | | **82%** | |
| McCarthy et al.  (2006) | **Y** | **Y** | **Y** | **Y** | **Y** | **CT** | **Y** | **Y** | **Y** | **CT** | **Y** | | **82%** | |
| Ouyang et al.  (2010) | **Y** | **Y** | **Y** | **Y** | **Y** | **Y** | **Y** | **Y** | **Y** | **Y** | **Y** | | **100%** | |
| Raspa et al.  (2014) | **Y** | **Y** | **Y** | **Y** | **Y** | **Y** | **Y** | **Y** | **Y** | **Y** | **Y** | | **100%** | |
| Wheeler et al.  (2008) | **Y** | **Y** | **Y** | **Y** | **Y** | **Y** | **Y** | **Y** | **Y** | **Y** | **Y** | | **100%** | |
| **Y: Yes N: No CT: Can’t tell** | | | | | | | | | | | | | |  |

**Q1:** Did the study address a clearly focused issue? **Q2:** Did the authors use an appropriate method to answer their question? **Q3:** Were the subjects recruited in an acceptable way? **Q4:** Were the measures accurately measured to reduce bias? **Q5:** Were the data collected in a way that addressed the research issue? **Q6:** Did the study have enough participants to minimize the play of chance? **Q7:** How are the results presented and what is the main result? **Q8:** Was the data analysis sufficiently rigorous? **Q9:** Is there a clear statement of findings? **Q10:** Can the results be applied to the local population? **Q11:** How valuable is the research?

**Table S2.** Quality assessment of the longitudinal study using CASP Cohort Study Tool

| Study | | Q1 | | Q2 | Q3 | Q4 | Q5a | Q5b | Q6a | Q6b | Q7 | Q8 | Q9 | Q10 | Q11 | Q12 | Total Quality  Assessment |
| --- | --- | --- | --- | --- | --- | --- | --- | --- | --- | --- | --- | --- | --- | --- | --- | --- | --- |
| Hauser et al. (2014) | | **Y** | | **Y** | **Y** | **Y** | **CT** | **CT** | **Y** | **Y** | **Y** | **Y** | **Y** | **Y** | **Y** | **Y** | **85%** |
| Fielding-  Gebhardt  et al. (2020) | | **Y** | | **Y** | **Y** | **Y** | **CT** | **CT** | **Y** | **Y** | **Y** | **Y** | **Y** | **Y** | **Y** | **Y** | **85%** |
|  |  | | **Y: Yes N: No CT: Can’t tell** | | | | | | | | | | | | | | |

**Q1:** Did the study address a clearly focused issue? **Q2:** Was the cohort recruited in an acceptable way? **Q3:** Was the exposure accurately measured to minimise bias? **Q4:** Was the outcome accurately measured to minimise bias? **Q5b:** Have the authors identified all important confounding factors? **Q5b:** b) Have they taken account of the confounding factors in the design and/or analysis? **Q6a:** Was the follow up of subjects complete enough? **Q6b:** Was the follow up of subjects long enough? **Q7:** What are the results of this study? **Q8:** How precise are the results? **Q9:** Do you believe the results? **Q10:** Can the results be applied to the local population? **Q11:** Do the results of this study fit with other available evidence? **Q12**: What are the implications of this study for practice?

**Table S3.** Quality assessment of the qualitative studies using CASP Qualitative Research Tool

| CASP Criteria | Brady et al. (2006) | Jones et al. (2023) | Luermans et al.  (2023) | Van Remmerden et al. (2019) | Visootsak et al.  (2012) |
| --- | --- | --- | --- | --- | --- |
| 1.Was there a clear statement of the aims of the research? | **Y** | **Y** | **Y** | **Y** | **Y** |
| 2. Is a qualitative methodology  appropriate? | **Y** | **Y** | **Y** | **Y** | **Y** |
| 3. Was the research design  appropriate to address the aims of  the research? | **Y** | **Y** | **Y** | **Y** | **Y** |
| 4. Was the recruitment strategy  appropriate to the aims of the  research? | **Y** | **Y** | **Y** | **CT** | **CT** |
| 5. Was the data collected in a way that addressed the research issue? | **Y** | **Y** | **Y** | **Y** | **Y** |
| 6. Has the relationship between  researcher and participants been  adequately considered? | **N** | **N** | **CT** | **CT** | **N** |
| 7. Have ethical issues been taken  into consideration? | **Y** | **Y** | **Y** | **Y** | **Y** |
| 8. Was the data analysis sufficiently rigorous? | **Y** | **Y** | **Y** | **Y** | **Y** |
| 9. Is there a clear statement of findings? | **Y** | **Y** | **Y** | **Y** | **Y** |
| 10. How valuable is the research? | **Y** | **Y** | **Y** | **Y** | **Y** |
| Total quality assessment | **90%** | **90%** | **90%** | **80%** | **80%** |
| **Y: Yes N: No CT: Can’t tell** | | | | | |
